# Comparison of the efficacy and safety of antibiotic treatment and appendectomy for acute uncomplicated appendicitis: a systematic review and meta-analysis

**DOI:** 10.1186/s12893-023-02108-1

**Published:** 2023-07-24

**Authors:** Hongxia Xu, Shaohui Yang, Jiankun Xing, Yan Wang, Weiqiang Sun, Lingyan Rong, Huihui liu

**Affiliations:** Department of Clinical Pharmacy, Wendeng Hospital of Traditional Chinese Orthopedics and Traumatology of Shandong Province, No.1 Fengshan Road, Wendeng District, Weihai City, 264400 Shandong Province China

**Keywords:** Antibiotic, Appendectomy, Acute uncomplicated appendicitis, Meta-analysis

## Abstract

**Objective:**

This meta-analysis aimed to compare the efficacy and safety of antibiotic treatment and appendectomy for acute uncomplicated appendicitis.

**Methods:**

We searched the randomized controlled studies (RCTs) comparing appendectomy with antibiotic treatment for uncomplicated acute appendicitis in the electronic database including Pubmed, Embase, Cochrane, Web of Science, CNKI, VIP, and WanFang. The primary outcomes included complication-free treatment success at 1 year, complications, surgical complications, and the complicated appendicitis rates. Secondary outcomes included negative appendicitis, length of hospital stay, the quality of life at 1 month, and the impact of an appendicolith on antibiotic therapy.

**Results:**

Twelve randomized controlled studies were included. Compared with surgery group, the antibiotic group decreased the complication-free treatment success at 1 year (RR 0.81; 95% CI 0.73–0.91; z = 3.65; *p* = 0.000). Statistically significance was existed between antibiotic group and surgical group with both surgical types(open and laparoscopic) (RR 0.43; 95% CI 0.31–0.58; z = 5.36; *p* = 0.000), while no between the antibiotic treatment and laparoscopic surgery (RR 0.72; 95% CI 0.41–1.24; z = 1.19; *p* = 0.236). There was no statistically significant differences between two groups of surgical complications (RR 1.38; 95% CI 0.70–2.73; z = 0.93; *p* = 0.353), the complicated appendicitis rate (RR 0.71; 95% CI 0.36–1.42; z = 0.96; *p* = 0.338), negative appendectomy rate (RR 1.11; 95% CI 0.69–1.79; z = 0.43; *p* = 0.670), duration of hospital stay (SMD 0.08; 95%CI -0.11-0.27; z = 0.80; *p* = 0.422), and quality of life at 1 month (SMD 0.09; 95%CI -0.03-0.20; z = 1.53; *p* = 0.127). However, in the antibiotic treatment group, appendicolith rates were statistically higher in those whose symptoms did not improve (RR 2.94; 95% CI 1.28–6.74; z = 2.55; *p* = 0.011).

**Conclusions:**

Although the cure rate of antibiotics is lower than surgery, antibiotic treatment is still a reasonable option for patients with uncomplicated acute appendicitis who do not want surgery without having to worry about complications or complicating the original illness.

**Supplementary Information:**

The online version contains supplementary material available at 10.1186/s12893-023-02108-1.

## Introduction

Acute appendicitis with the incidence of approximate 1/1000 person-years, which affect 8 million annually, is the most common reason for emergency abdominal surgery [1]. The etiology of acute appendicitis is generally fecal residue or lymphoid tissue proliferation blocking the appendiceal lumen, resulting in high pressure in the lumen and damage to the integrity of the mucosa [2, 3]. Acute appendicitis is classified as either uncomplicated or complicated acute appendicitis [4]. Though the definition of them varies among studies [5], generally the uncomplicated acute appendicitis may absence of perforation, abscess or peritonitis and may or may not include non-perforated gangrenous or a fecalith [6–9].

For many years, appendectomy has been recommended for acute uncomplicated appendicitis [10]. Complication after appendectomy, such as wound infection, intestinal adhesions, incisional hernias and so on, are between 2% and 23% [11–13]. Laparoscopic appendectomy is preferred to an open approach with a lower incidence of complications [5]. However, open appendectomy is still used if the appendix has burst or if access is difficult [14].

As early as 1950, people began to try non-surgical treatment for acute simple appendicitis, but it was not generally accepted [15]. For a long time, it was believed that every uncomplicated appendicitis would ultimately progress into a complicated appendicitis [16]. However, growing evidence shows that uncomplicated and complicated acute appendicitis have followed different epidemiological trends which may be treated differently [17, 18]. This has increased new interest for the use of antibiotics in the uncomplicated acute appendicitis. Recently, an increasing amount of evidence supports the use of antibiotics instead of surgery for treating patients with uncomplicated acute appendicitis [19–22]. The cure rate of the uncomplicated acute appendicitis treated with antibiotics is generally 73–88% [7, 8, 23], but over time, within five years it is generally 54–61% [24, 25], which is lower than that after surgery. The complications induced by antibiotic therapy were less than 6.5% and 4.5–24.4% in the appendectomy group [24, 26]. Although cure rate in the antibiotic group was lower, but about half of the participants preferred antibiotics for avoiding surgery [27], and people under the high risk of appendectomy because of comorbidities had to choose conservative treatment [28]. The appendix has a certain immune function and can store intestinal flora, both of which affect the progression of desease, such as cancer [29], so the significance of appendix retention is increased. And during the COVID-19 pandemic, antibiotic treatment of uncomplicated appendicitis showed attraction [30].

However, antibiotic treatment of uncomplicated appendicitis is still limited by conflicting results coming from studies [31, 32] and some guidelines [5, 28, 33, 34]. This inconsistency may be largely due to a lack of evidence. Recently, the new literature on antibiotic therapy has made progress [7, 9, 35, 36]. Meanwhile, we found that different outcomes of the treatment of uncomplicated appendicitis between randomized and non-randomized controlled trials [20]. And we also found that the meta-analyses that included all randomized trials were rare, and the number of relevant literature is small [22, 37]. Additionally, although more randomized controls were included, semi-randomized controlled trials or complicated appendicitis were included [21, 38].

To help patients better choose their treatment solutions, we made a systematic review and meta-analysis that only RCTs studied on uncomplicated appendicitis included to compare the efficiency of antibiotic treatment with appendectomy, which included indicators of cure rates, complications, and examining whether delayed surgery with antibiotics treatment resulted in a higher rate of complicated appendicitis, et al., to fully discuss the advantages and disadvantages of both treatment options for clinic patients.

## Methods

### Registration

The systematic review and meta-analysis were carried out in line with the recommendations of the Preferred Reporting Items for Systematic Reviews and Meta-Analyses (PRISMA) guidelines [39], and was specified in a registered protocol CRD42022374759.

### Search strategy

Seven databases, including Pubmed, Embase, Cochrane, Web of Science, CNKI, VIP, and WanFang were searched from their inception to May 2022.

All stages of study identification, selection, quality assessment and data abstraction were carried out independently by 2 reviewers (Hongxia Xu and Shaohui Yang). Any discrepancies were resolved by consulting a third reviewer (Jiankun Xing). The search strategy consisted of medical subject headings (MeSH),including appendicitis, appendectomy, anti-bacterial agents and randomized controlled trial ,and text words. For example, the search strategy in Pubmed was as followed:(Appendicitis [Mesh] OR Ruptured Appendicitis [Title/Abstract] OR Appendicitis, Ruptured [Title/Abstract] OR Perforated Appendicitis [Title/Abstract]) OR Appendicitis, Perforated [Title/Abstract]) AND (Appendectomy [Mesh] OR Appendectomies [Title/Abstract]) AND (Anti-Bacterial Agents [Mesh] OR Agents, Anti-Bacterial [Title/Abstract] OR Anti Bacterial Agents [Title/Abstract] OR Antibacterial Agents [Title/Abstract] OR Agents, Antibacterial [Title/Abstract] OR Antibacterial Agent [Title/Abstract] OR Agent, Antibacterial [Title/Abstract] OR Anti-Bacterial Compounds [Title/Abstract] OR Anti Bacterial Compounds [Title/Abstract] OR Compounds, Anti-Bacterial [Title/Abstract] OR Anti-Bacterial Agent [Title/Abstract] OR Agent, Anti-Bacterial [Title/Abstract] OR Anti Bacterial Agent [Title/Abstract] OR Anti-Bacterial Compound [Title/Abstract] OR Anti Bacterial Compound [Title/Abstract] OR Compound, Anti-Bacterial  [Title/Abstract] OR Bacteriocidal Agents [Title/Abstract] OR Agents, Bacteriocidal [Title/Abstract] OR Bacteriocidal Agent [Title/Abstract] OR Agent, Bacteriocidal  [Title/Abstract] OR Bacteriocide[Title/Abstract] OR Bacteriocides[Title/Abstract] OR Anti-Mycobacterial Agents [Title/Abstract] OR Agents, Anti-Mycobacterial [Title/Abstract] OR Anti Mycobacterial Agents [Title/Abstract] OR Anti-Mycobacterial Agent [Title/Abstract])) OR Agent, Anti-Mycobacterial [Title/Abstract] OR Anti Mycobacterial Agent [Title/Abstract] OR Antimycobacterial Agent [Title/Abstract] OR Agent, Antimycobacterial [Title/Abstract] OR Antimycobacterial Agents  [Title/Abstract] OR Agents, Antimycobacterial [Title/Abstract] OR Antibiotics [Title/Abstract] OR Antibiotic [Title/Abstract]) AND (Randomized Controlled Trial  [Publication Type] OR rct) AND (1000/1/1:2022/5/31 [pdat]) AND (1000/1/1:2022/5/31 [pdat]) Filters: from 1000/1/1–2022/5/31.

The search strategies of databases were shown in Supplementary Table 1.

### Study selection criteria

1. Studies including people who diagnosed as uncomplicated appendicitis. In this article, uncomplicated appendicitis defines as absence of perforation, abscess, peritonitis, fecalith, perforated gangrenous or phlegmonous.
2. Only RCTs were included.
3. Only English and Chinese literatures (only included in the core above journals) were included.

### Risk of bias assessment

The risk of bias for the studies enrolled in the systematic review and meta-analysis was assessed according to the Cochrane handbook for systematic reviews of interventions [40], using the Cochrane risk of bias tool for RCTs.

### Outcome measures

#### The primary outcome measure

1. **Cure rate of complication-free treatment at 1 year**: 1-year cure rate without complications

In antibiotic treatment group, no recurrence, no moderate or serious adverse events required hospitalization, and in appendectomy treatment group, there were no post-operative complications.

2. **Total complications**

The complications in the appendectomy group were defined as postoperative complications, while in antibiotic group were adverse events requiring hospitalization.

3. **Complications after appendectomy**

The patients in the appendectomy group and in the antibiotic group who needed surgical had postoperative complications.

4. **The rate of complicated appendicitis formed after treatments**

If antibiotics delayed the initial treatment of uncomplicated appendicitis, the disease was defined as complicated appendicitis was found after antibiotic therapy.

#### The secondary outcome measure

1. Negative appendicitis

Negative appendicitis was defined as non-appendicitis was found after appendectomy.

2. Length of hospital stay
3. Quality of life after treatments
4. The effect of appendicoliths on the effectiveness of antibiotic treatment

In the antibiotic group, appendicolith rate was compared between patients whose symptoms improved and not.

### Data extraction

The data extraction was performed by two independent authors (Hongxia Xu and Shaohui Yang), and a third author (Jiankun Xing) adjudicated discordant assessments.

### Statistical analysis

Stata 16 was applied for data analysis. Heterogeneity of the results across studies was assessed using Higgins’ *I*^*2*^ and chi-square tests. A *p*-value of chi-square test less than 0.05 with an *I*^*2*^ value of greater than 50% was considered indicative of substantial heterogeneity.

Fixed-effects model was implemented if statistically significant heterogeneity was absent. Otherwise, a random-effects model was used for meta-analysis if statistically significant heterogeneity was found.

## Results

### Literature search, study selection, and characteristic

**Fig. 1:**
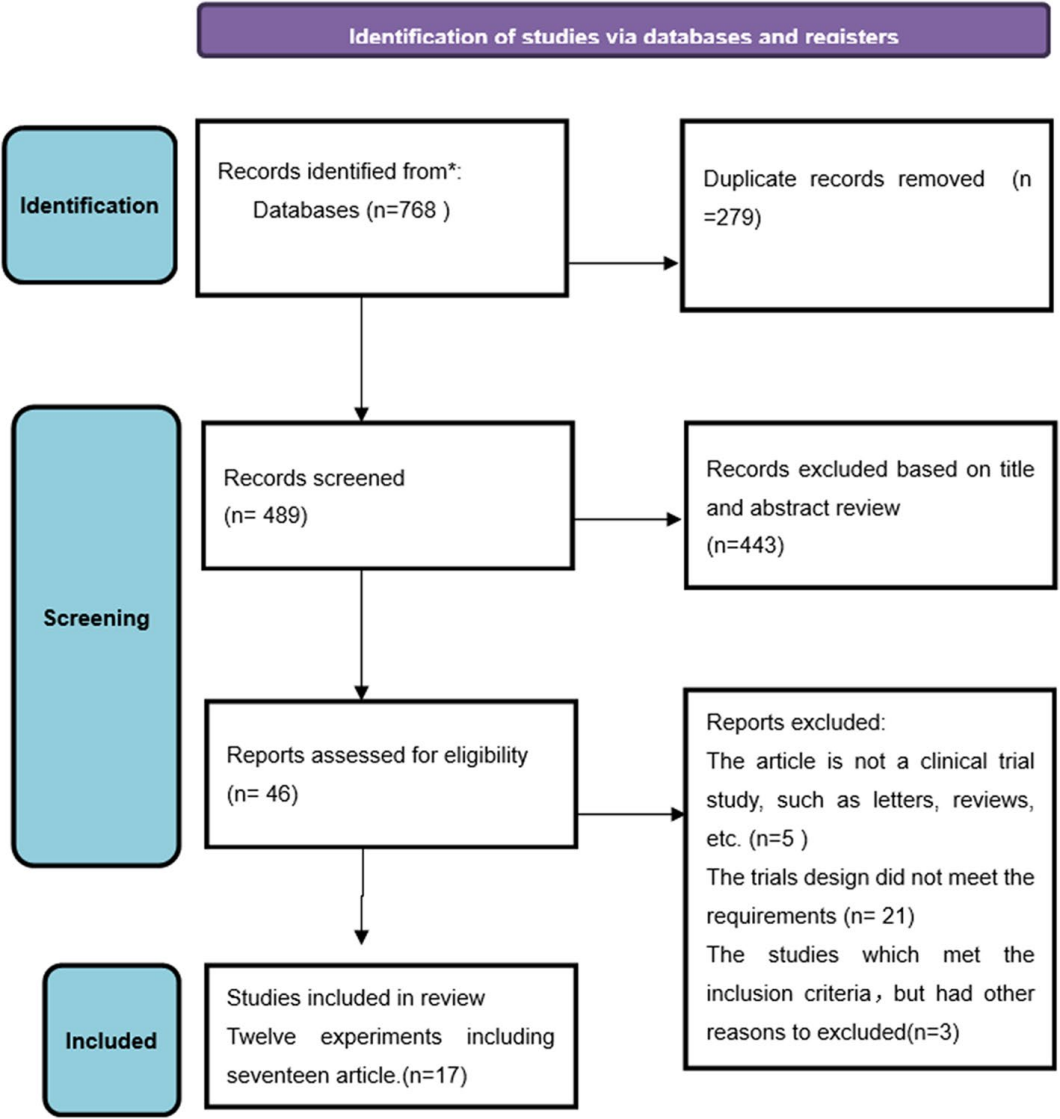
Flow diagram of literature screening and selection process

A total of 768 references were identified through data base searching (Fig. 1). The inclusion criteria were met by 20 articles. However, three studies which met the inclusion criteria were excluded after closer review: one is a quasi-randomization trial [41], one lacks evidence of randomization [42], and the third was retracted after publication [43]. So finally 17 articles were included [6–9, 23–26, 35, 36, 44–50]. Salminen 2018 [24], Haijanen 2019 [50], Sippola 2017 [48], and Sippola 2020 [49] were follow-up trial studies of Salminen 2015 [8]. Patkova 2020 [25] is a follow-up study to the Svensson2015 [46] trial. Although the number of articles included was 17, there were 12 RCTs in this study. The characteristics of included RCTs were list in Table 1.

**Table 1:** Characteristics of included RCTs

Number	Follow-up test	Test type	Included population	Diagnostic method	Antibiotic group	Operation group	Outcome indicators
Vons 2011 [23]		RCT,multicenter(6 centers)	*n* = 239 adult:>18 years;uncomplicated appendicitis	CT	*n* = 120 amoxicillin plus clavulanic acid 3 g ,iv 48 h,oral amoxicillin plus clavulanic for 8 days.	*n* = 119 a McBurney incision or laparoscopy	primary endpoint: occurrence of peritonitis within 30 days of initial treatment. Secondary endpoints: 1.the number of days with a postintervention visual-analogue-scale pain score ≥ 4. 2. length of hospital stay. 3.absence from work. 4.incidence of complications other than peri tonitis within 1 year. 5.recurrence of appendicitis after antibiotic treatment
Salminen 2015 [8]	Salminen 2018 [24, Haijanen 2019 [50], Sippla 2017, Sippla 2020	RCT,multicenter(6 centers)	*n* = 530 adult:aged 18–60 years.uncomplicated appendicitis.	CT	*n* = 257 Ertapenem 1 g/d IV for 3 days, followed by 7 days of oral levofloxacin (500 mg once daily) and metronidazole(500 mg 3 times per day).	*n* = 273 mostly Open appendectomy,laparoscopic appendectomy 5.5%.	primary endpoint: successful treatment.antibiotic group. Secondary endpoints: 1.overall post intervention complications. 2.late recurrence (after 1 year) of acute appendicitis after conservative treatment 3.length of hospital stay 4.sick leave 5.postintervention pain scores 6.treatment costs
Otero 2022 [6]		RCT,multicenter(3 centers)	*n* = 39 children: aged 6–17 years. uncomplicated appendicitis.	ultrasound and/or computed tomography (CT)	*n* = 20 piperacillin/tazobactam iv for 24–48 h, followed by 10 days of oral ciprofloxacin and metronida-zole	*n* = 19 laparoscopic appendectomy	Primary endpoint: (1) one-year success rate of antibiotics-alone. (2) QOL measures assessed 1 month post discharge. Secondary endpoints: length of stay and readmission rate.
Svensson 2015 [46]	Patkova 2020	Pilot RCT(2 centers).	*n* = 50 children: aged 5–15 years. acuteappendicitis,exclusing perforated appendicitis on the basis of generalized peritonitis、appendiceal mass.	ultrasound and/or computed tomography (CT)	*n* = 24 meropenem (10 mg/kg × 3 per 24 h) and metronidazole (20 mg/kg × 1 per 24 h) IV for at least 48 h,followed by 8 days of oral ciprofloxacin (20 mg/kg×2 per 24 h) andmetronidazole (20 mg/kg × 1 per 24 h).	*n* = 26 laparoscopic appendectomy	primary endpoint: resolution of symptoms without significant complications. Secondary endpoints: 1.the time from randomization to discharge. 2.complications 3.recurrent appendicitis within 1 year 4.cost of treatment
Styrud 2006 [26]		RCT,multicenter(6 centers)	*n* = 252 adult, men:18–50 years. Excluded were patients with suspicion of perforation of the appendix.	laboratory results,the other diagnisis not clear.but The frequency of appendicitis was 97% in the surgery group.	*n* = 128 2 days of i.v. cefotaxime 2 g 12 hourly, and tinidazole 0.8 g daily,and received oral treatment with ofloxacin 200 mg twice daily, and tinidazole 500 mg twice daily for 10 days.	*n* = 124 open or laparoscopically	1.The cure rate of antibiotic group and operation group. 2.Recurrence rate of antibiotic group within one year 3.the time off work 4.Sick leave 5.Hospital stay
Peter O’Leary 2021 [35]		RCT	*n* = 186 adult:>16 years; uncomplicated appendicitis	< 45 years,US +/- MRI;>45 years,CT	*n* = 91 co-amoxiclav,1.2 g,IV 3 times daily until there was a clinical improvement followed by 5 days of oral co-amoxiclav(625 mg 3 times a day orally for 5 days).	*n* = 89 laparoscopic appendectomy	primary endpoint: evaluated the success rate for acute uncomplicated appendicitis at 1-year follow-up. Secondary endpoints: 1.quality of life 2.cost 3.length of stay 4.sickness days
Hall 2020		RCT	*n* = 54 children: aged 3–16 years. uncomplicated appendicitis.	clinical and laboratory parameters、adiologicalassessment.In this trial, just 28% of participants received an ultrasound.	*n* = 27 a minimum of 24 h broad spectrum intravenous antibiotics, After the initial 12-hour period of NBM(nil by mouth), oral intake was advanced as tolerated, received a total of 10 days antibiotics following randomisation。	*n* = 27 open or laparoscopic appendicectomy	primary endpoint: the recruitment rate into the trial. Secondary endpoints: 1.Performance of study procedures. 2. Willingness of parents, children and surgeons to take part in a randomised study comparing operative versus non-operative treatment and identify anticipated recruitment rate. 3. Clinical outcomes of trial treatment pathways.
Ceresoli 2018		prospective RCT	adult:aged 18–65 years. uncomplicated appendicitis.	appendicitis inflammatory response (AIR) score with the adjunct of ultrasound.	*n* = 19 intravenous administration of 1 g of Ertapenem once a day for 3 days during hospitalization and further administration of amoxicillin/clavulanate 1 g every 8 h for 5 days.	*n* = 22 laparoscopic appendectomy	primary endpoint: the success rate of the treatment. Secondary endpoints: 1.complication rate 2.negative appendectomy rate 3. long-term negative outcomes within a year 4.Length of hospital stay 5. work absence
Talan 2016		Pilot RCT	*n* = 30 children(*n* = 1) + adult: aged > 5 years. uncomplicated appendicitis	CT and/or ultrasonography:CT with intravenous contrast in adults and ultrasonography in persons younger than 18 years, followed by CT if indicated.	*n* = 16 intravenous ertapenem for 2 day,followed 8-day supply of an oral antibiotic regimen of cefdinir and metronidazole.	*n* = 14 open or laparoscopic appendectomy	primary endpoint: the one-month major complication rate. Secondary endpoints: 1. appendectomy rate in the antibiotics-first group 2.quality of life 3. days unable to perform normal activities and work or school 4.days of analgesic use 5. pain scores 6. total hours in the ED and hospital at the initial visit from triage until ED or hospital discharge 7. total hours in the hospital (including the ED) through 1 month 8. Alvarado scores on day 1 for both groups and day 2 for antibiotics-first participants 9. hospital charges.
Sajjad 2021 [36]		RCT	*n* = 180 children: aged 5–15 years. uncomplicated appendicitis.	Ultrasound (USG) findings、PAS score、laboratory results	*n* = 90 intravenous meropenem (10 mg/kg/dose intravenous infusion 8 hourly) and metronidazole (20 mg/kg/day intravenous divided doses 8 hourly) for at least 48 h. Followed oral ciprofloxacin (10 mg/kg/dose twice daily) and metronidazole (20 mg/kg/day two divided doses) for another 8 days	*n* = 90 open appendectomy	1.successful cute rate 2.Total leukocyte count 3. C reactive protein
Eriksson 1995 [47]		RCT	*n* = 40 adult: aged 18–75 years, acute appendicitis.	ultrasonography and laboratory tests	*n* = 20 Cefotaxime 2 g 12 hourly and tinidazole 800 mg daily were given for 2 days, followed oral treatment with ofloxacin 200 mg twice daily and tinidazole 500 mg twice daily for 8 days.	*n* = 20 open appendectomy	1.Mean concentration of C-reactive protein during hospitalization and at 30 days of follow-up 2.Mean total white blood cell count during hospitalization and at 30 days of follow-up 3.Pain record during hospitalization and at 30 days of follow-up 4.cute rate
CODA collaborative 2020 [7]		RCT, multicenter(25 centers)	*n* = 1552 adult	computed tomography (CT) alone or in combination with ultrasonography or magnetic resonance imaging	*n* = 776 received an intravenous formulation for at least 24 h, followed by pills, for a 10-day total course. Clinical teams selected antibiotics from Surgical Infection Society and Infectious Diseases Society of America guidelines for intraabdominal infections.	*n* = 776 laparoscopic(96%) and open appendectomy	primary endpoint: 30-day health status Secondary endpoints: 1.patient-reported resolution of symptoms 2.Visits to the emergency department or urgent care clinic for related symptoms 3. days in the emergency department or hospital related to appendicitis symptoms or treatment-related complications 4.days of missed work for the participant and the caregiver

In addition to data directly extracted from the articles, some data was deduced from graphs, or proportions (Peter O’Leary 2021) [35], some was calculated from other data given in the article (CODA Collaborative 2020, Vons 2011, Styrud 2006.) [7, 23, 26], and some were derived from composite data, such as data without an appendicolith representing uncomplicated appendicitis (CODA Collaborative 2020.) [7].

If the format of the measure is not mean and standard deviation, manual calculation into mean (standard deviation) was used if it is the mean (95% CI), and calculation software is applied if it is the median [51, 52].

### Risk of Bias

**Fig. 2:**
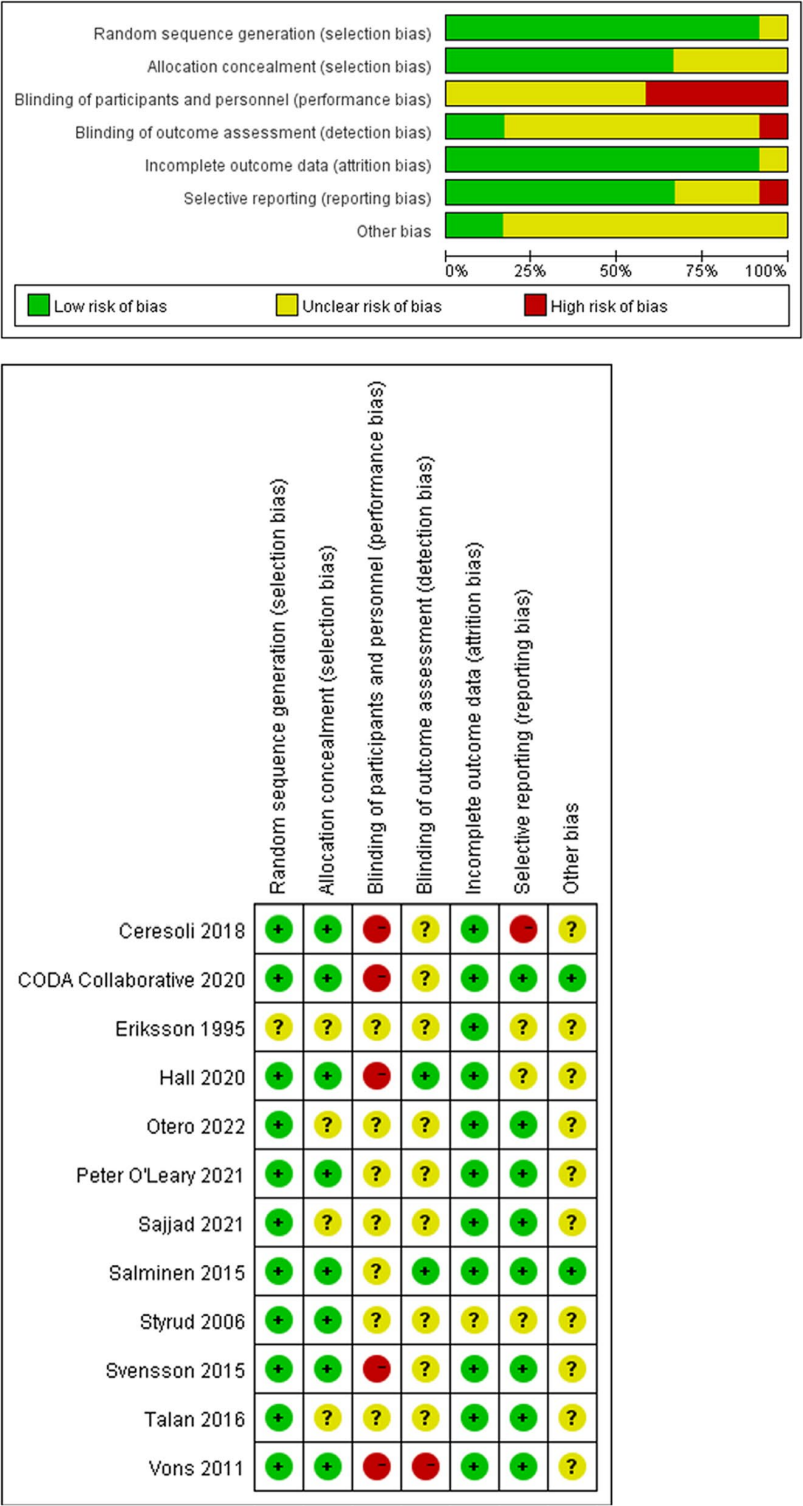
Risk of bias graph

Twelve trials were included for quality evaluation, which used revman 5.3 ( Fig. 2).

### Publication bias

#### Total complications

A total of 11 RCTS were included in the “complications” analysis, and the Egger’s test result was that *p* = 0.952 was greater than 0.05, and there was no evidence for publication bias in the articles. See Annex 1 for details.

#### Complications after appendectomy

A total of 11 RCTS were included in the “surgical complications” analysis, and the Egger’s test result was that *p* = 0.838 was greater than 0.05, and there was no evidence for publication bias between the articles. See Annex 2 for details.

#### The rate of complicated appendicitis formed after treatments

A total of 10 RCTS were included in the “The Complimentary Rate” analysis, and the result of Egger’s test was that *p* = 0.177 was greater than 0.05, so there was no evidence of publication bias between the articles. See Annex 3 for details.

#### Negative appendicectomies

A total of 10 RCTs were included in the “The Complimentary Rate” analysis, and the Egger’s test result was that *p* = 0.475 was greater than 0.05, and there was no evidence for publication bias between the articles. See Annex 4 for details.

## Conclusion

### Leading indicators

#### Cure rate of complication-free treatment at 1 year

**Fig. 3:**
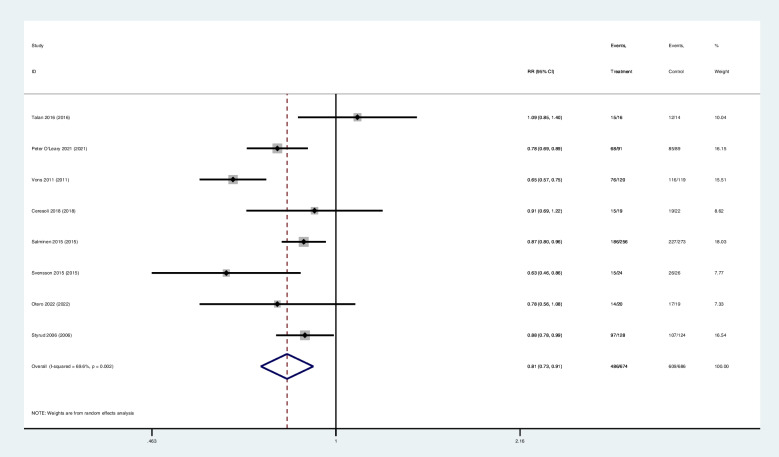
Forest diagram of complication-free treatment success at 1year

Eight studies reported the results of treatment success at 1year. The complication-free treatment success rate of antibiotic group (69.4%,468/674) was inferior to that of surgery group (88.8%,609/686) (RR 0.81; 95% CI 0.73–0.91; z = 3.65; *p* = 0.000), has a significant difference (Table 2) (Fig. 3).

**Table 2:** Outcomes of the primary and secondary indicators

Indicators	Antibiotic group	Surgery group	RR (95% CI)	z	*p*
Cure rate of complication-free treatment at 1 year	69.4%(468/674)	88.8%(609/686)	0.81(0.73–0.91)	3.65	0.000 < 0.05
Total complications	3.9%(50/1270)	9.5%(119/1257)	0.43(0.31–0.58)	5.36	0.000 < 0.05
	4.8%(30/631)	14.5%(91/626)^a^	0.34(0.23–0.49)	5.55	0.000 < 0.05
	3.1%(20/639)	4.4%(28/631)^b^	0.72(0.41–1.24)	1.19	0.236
Complications after appendectomy	9.5%(119/1257)	11.9%(47/394)	1.38(0.70–2.73)	0.93	0.353
Therate of complicated appendicitis formed after treatments	5%(63/1264)	9.9%(128/1289)	0.71(0.36–1.42)	0.96	0.338
The rate of appendectomy with non-appendicitis	4.2%(30/721)	3.7%(27/732)	1.11(0.69–1.79)	0.43	0.670
Length of hospital stay	NA	NA	0.08(-0.11-0.27)^c^	0.80	0.422
Quality of life after treatments	NA	NA	0.09(-0.03-0.20)^c^	1.53	0.127
The effect of appendicoliths on the effectiveness of antibiotic treatment	38.1%(101/265)^d^	18.9%(140/740)^e^	2.94(1.28–6.74)	2.55	0.011 < 0.05

*NA *Not Available

^a^Open surgery

^b^Laparoscopic surgery

^c^SMD (95%CI)

^d^The appendicolith rate of the patients whose symptoms were not improved in the antibiotic treatment group

^e^The appendicolith rate of the patients whose symptoms were improved in the antibiotic treatment group

#### Total complications

Eleven studies reported data on complications, of which five studies were mostly laparoscopic surgery, six mostly open surgery.

**Fig. 4:**
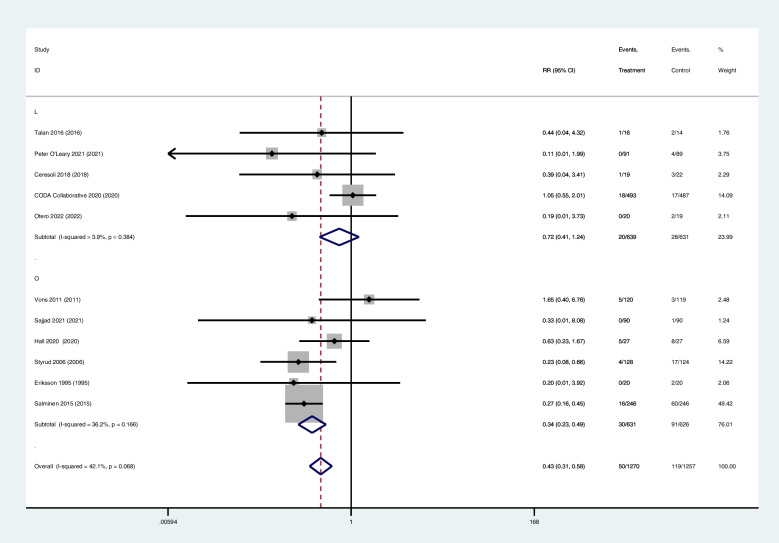
Forest diagram of complications

The complications between antibiotic group(3.9%, 50/1270) and surgical group with both surgical types (open and laparoscopic) (9.5%, 119/1257) had statistically significant (RR 0.43; 95% CI 0.31–0.58; z = 5.36; *p* = 0.000).Subgroup analyses revealed significant differences between antibiotic treatment (4.8%, 30/631) and open surgery (14.5%, 91/626) (RR 0.34; 95% CI 0.23–0.49; z = 5.55; *p* = 0.000). However, there was no statistically significant between antibiotic treatment (3.1%, 20/639) and laparoscopic surgery (4.4%, 28/631) (RR 0.72; 95% CI 0.41–1.24; z = 1.19; *p* = 0.236). (Table 2) (Fig. 4).

#### Complications after appendectomy

**Fig. 5:**
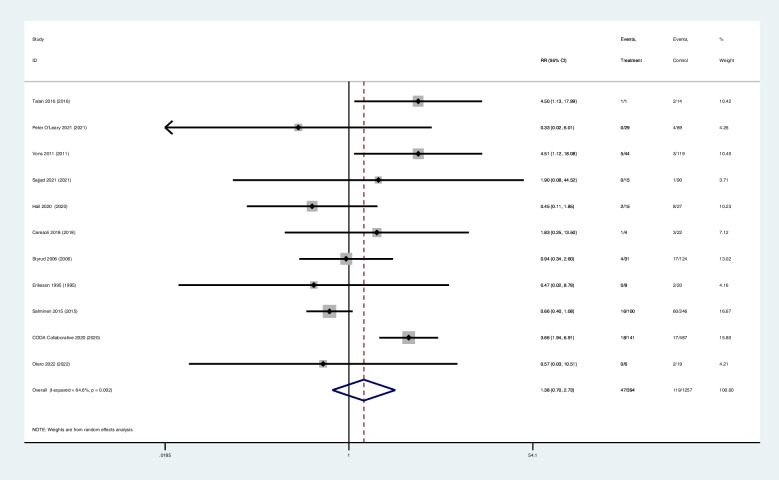
Forest diagram of postoperative complications

No statistically significant differences were found between antibiotic group who eventually underwent appendectomy (9.5%, 119/1257) and surgical group (11.9%, 47/394) for complications (RR 1.38; 95%CI 0.70–2.73; z = 0.93; *p* = 0.353), though complications rate of antibiotic group is higher than surgical group (Table 2) (Fig. 5).

#### The rate of complicated appendicitis formed after treatments

**Fig. 6:**
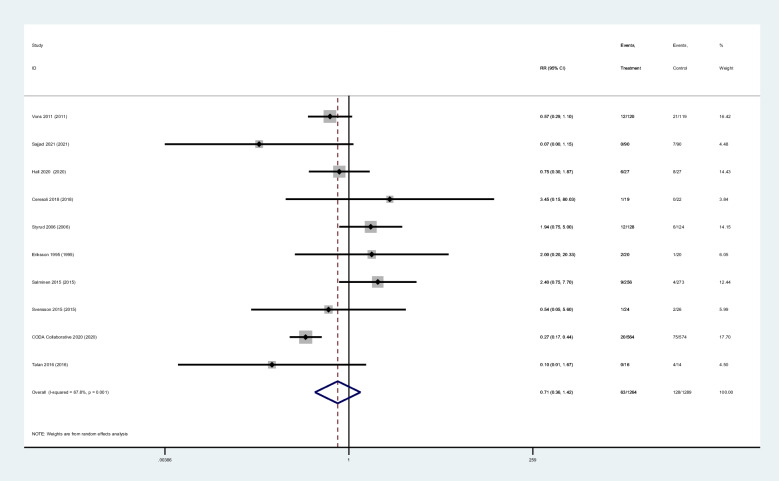
Forest diagram of the complicated appendicitis rate

Ten studies reported the incidence of complicated appendicitis undergoing surgery in both antibiotic and surgical groups. Compared to surgical groups, the complicated appendicitis rate was lower in antibiotic groups (5%, 63/1264; 9.9%, 128/1289), however there is no statistically significant differences between two groups (RR 0.71; 95% CI 0.36–1.42; z = 0.96; *p* = 0.338) (Fig. 6).

### Secondary indicators

#### Negative appendicectomies

Ten studies reported negative appendectomy in both groups.

The rates of negative appendectomy in antibiotic group after antibiotic treatment failure and surgical group were equivalent (4.2%, 30/721; 3.7%, 27/732), the antibiotic group was slightly higher, but there is no statistically significant differences between two groups (RR 1.11; 95% CI 0.69–1.79; z = 0.43; *p* = 0.67) (Table 2). See Annex 5 for details.

#### Length of hospital stay

The length of hospital stay was reported in six studies. The duration of hospital stay did not differ significantly between the antibiotic and surgical group (SMD 0.08; 95%CI -0.11-0.27; z = 0.80; *p* = 0.422) (Table 2). See Annex 6 for details.

#### Quality of life after treatments

The literature that could be analyzed for the quality of life were small and only 3 papers were included for the quality of life at 1 month. The quality of life did not differ significantly between the antibiotic group and surgical group at one month after treatment (SMD 0.09; 95%CI -0.03-0.20; z = 1.53; *p* = 0.127) (Table 2). See Annex 7 for details.

#### The effect of appendicoliths on the effectiveness of antibiotic treatment

A total of four studies reported the effect of an appendicolith on the effectiveness of antibiotics. The appendicolith rate was about twice in patients whose symptoms did not improved(38.1%, 101/265)than those whose symptoms improved (18.9%, 140/740).There was a statistically significant difference between two groups(RR 2.94; 95% CI 1.28–6.74; z = 2.55; *p* = 0.011) (Table 2). See Annex 8 for details.

## Discussion

Comparing treatment success between the antibiotic and surgical groups, the complication-free cure rate is more objective. The cure rate of antibiotic group in this study is 69.4%, which is consistent with the previous study [20, 53]. In comparision with surgical, the cure rate of the antibiotic group is significantly lower, suggesting that it may not be the optimum treatment for uncomplicated acute appendicitis only considering of recurrence. However, there were certain patients who were not fit or willing for surgery. Bom WJ et al. [27] found that about half of the participants preferred antibiotic treatment for avoiding surgery and would accept a recurrence risk of more than 50% within 1 year. However, participants who prefer surgery for radical treatment of appendicitis, may tolerate a recurrence risk of no more than 10% when treated with antibiotics.

In general, the complications in the antimicrobial therapy group were lower than those in the surgical group. However, the surgical procedures included open and laparoscopic surgery, and the complications of open surgery were higher than those of laparoscopic surgery [54]. Complications of antibiotic therapy were 1.5-8.2% [24, 36, 42], and 1%~3% of laparoscopic operation [55–57]. According to this study, the complications rates between laparoscopically operated people (4.4%) and antibiotic treated people (3.1%) were similar. With the introduction of laparoscopic surgery, the complications of surgery are greatly reduced and comparable to conservative treatment, which is a significant advantage of laparoscopic appendectomy.

This study showed that patients who underwent surgery after failing antibiotic treatment had similar surgical complications rates as the surgical group (9.5% vs. 11.9%), suggesting that delaying the appendectomy due to antibiotic failure might not possibly result in a higher risk of postoperative complications. The previous study found that the rate of complicated appendicitis was lower in the antibiotics treatment than surgical group (2.7% vs. 12.3%) at 1 year. Antibiotics treatment does not increase the rate of complicated appendicitis [58], which is familiar with the present study(antibiotics treatment vs. surgery group :5% vs. 9.9%). Uncomplicated acute appendicitis treated with antibiotics first was safe and effective with no significant increase in the number of complicated acute appendicitis [5, 32, 59, 60]. Also, evidence suggests that spontaneous resolution of untreated, non-perforated appendicitis is common and the perforation can rarely be prevented [17]. However, the previous Meta-analysis study [20] showed the opposite conclusion because it used the number of surgical patients instead of all patients (which generally applied)of antibiotic group as a parameter. Additionally, it is possible that some patients may have had complicated appendicitis at first that was not diagnosed, as opposed to uncomplicated appendicitis progressing to complicated appendicitis due to antibiotic treatment failure. Therefore, the conclusion that uncomplicated appendicitis will progress to complicated appendicitis can only be overestimated.

Dozens of studies found that the incidence of negative appendectomies varies greatly from approximately 3.75–21% [57, 61, 62]. Normally, there are two reasons for such wide range as follows: (1)Preoperative imaging such as Computed Tomography(CT) and Ultrasound (US), has widespread used to greatly reduce the proportion of negative appendices in recent years. CT has been shown greater sensitivity and specificity than US for the diagnosis of appendicitis [63–65]; (2)Some considered completely normal appendicitis as a negative appendectomy, while the others included hyperplasia, atrophy and fibrosis. The present study applies the latter definition. The incidence of negative appendectomy was lower in both groups(antibiotics treatment vs. surgery group:4.2% vs. 3.7%), similar to another study [57]. In this meta-analysis, ten studies reported negative appendectomies, only one trials (Styrud2006) [26] did not explicit mention if preoperative imaging was used. Therefore, we should increase the accuracy of preoperative imaging diagnosis, and if US is difficult to clarify, CT, even Magnetic Resonance Imaging(MRI), can be added [66].

A majority of articles documented similar hospital stay with both treatment methods [22, 32, 37, 67], which is consistent with our study. However, some reported that the length of hospital stay in the antibiotics group was longer than that of the surgery group [38, 68], while others reported that the conservative group’s hospital stay was shorter than the surgery group [53].

This meta-analysis only analyzed the quality of life at 1 month and there was no significant difference. Podda M et al. concluded that the score of quality of life was significantly higher in patients with the appendectomy treatment at the 30-day, while the score was lower in the appendectomy group at the 1-year [69]. Minneci PC reported that patients who selected nonoperative management had high quality-of-life scores and remained satisfied with their health care decision at both 30 days and 1 year [65].

Dozens of studies have shown that presence of an appendicolith is associated with both an increased risk of antibiotic failure and recurrence [70–72], which is consistent with this study.

Appendiceal tumors were found in only 2 studies. Salminen2015 [8] reported that four patients had appendiceal tumors in surgical group and no appendiceal tumors were found in antibiotic group, while CODA Collaborative 2020 [7] reported seven and two respectively. Fewer appendiceal tumors were reported in the antibiotic group. The rate of misdiagnosis of appendiceal tumors was high, which is reported between0.7 and 2.5% [73–77]. Currently known risk factors for appendiceal tumors are age and complicated appendicitis [78, 79].

During relapse of failed treatment in the antibiotic group, antibiotics can be treated again. The previous study [44] reported that 2 patients successfully treated with antibiotics again when had recurrence appendicitis. Di Saverio S et al. pointed that a second attempt with antibiotic treatment could be a successful option for over 60% of patients who present with a recurrent episode of appendicitis at follow-up [80]. Poillucci G et al. found that 3.3% of patients who presented with a recurrence at follow-up were successfully treated with a further cycle of antibiotics [81]. Antibiotics treatment will not aggravate the progression of uncomplicated appendicitis. Thus, when the patient relapses after initial antibiotic treatment, antimicrobial therapy can be used again if the diagnosis of appendicitis is confirmed.

This study suggested the treatment of uncomplicated appendicitis included: it is necessary to increase the accuracy of preoperative imaging diagnosis to decrease the rate of appendectomy in non-appendicitis patients. If the patient is extremely concerned about the cure and recurrence rate of appendicitis, we recommend surgical intervention instead of antimicrobial therapy for them and laparoscopic surgery is recommended. If patients resist surgery and wish to be treated with antibiotics, they should be informed that the recurrence rate of antibiotics is significantly higher than surgery. However, there is no need to worry about uncomplicated appendicitis developing into complicated appendicitis. If patients had uncomplicated appendicitis with appendicoliths, surgery should be recommended.

There are still some limitations in this meta-analysis. The large difference in trial scale for inclusion articles may lead to result bias. Meanwhile, some outcome indicators were included in relatively few articles, which might have an impact on the outcome analysis.

## Conclusions

In this meta-analysis, we found strong evidence that antibiotics has a significantly lower complication-free cure rate than surgical treatment to the uncomplicated acute appendicitis, and the total complications of laparoscopic surgery are comparable to antibiotic treatment. Notably, patients who are particularly concerned about appendicitis recurrence should be cautious when choosing antibacterial drugs for the treatment of uncomplicated appendicitis. Patients who failed antibiotic treatment first and underwent surgery later could not possibly result in a higher risk of postoperative complications and the uncomplicated appendicitis does not develop to complicated appendicitis in this course, which made antibiotic treatment appealing for patients with uncomplicated acute appendicitis who do not want surgery without having to worry about complications or complicating the original illness. The negative appendectomy results depend on the preoperative imaging and the presence of an appendicolith is associated with an increased risk of antibiotic failure.

## Supplementary Information

**Additional file 1.**

## Data Availability

All data analyzed in this study are included in the references. Further information about the data can be accessed through the corresponding authors of the references.

## References

[1] Ferris M, Quan S, Kaplan BS (2017). The global incidence of appendicitis: a systematic review of population-based studies. Ann Surg.

[2] Stringer MD (2017). Acute appendicitis. J Paediatr Child Heath.

[3] Dzabic M, Bostrom L, Rahbar A (2008). High prevalence of an active cytomegalovirus infection in the appendix of immunocompetent patients with acute appendicitis. Inflamm Bowel Dis.

[4] Haijanen J, Sippola S, Grönroos J (2018). Optimising the antibiotic treatment of uncomplicated acute appendicitis: a protocol for a multicentre randomized clinical trial (APPAC II trial). BMC Surg.

[5] Di Saverio S, Podda M, De Simone B (2020). Diagnosis and treatment of acute appendicitis: 2020 update of the WSES Jerusalem guidelines. World J Emerg Surg.

[6] Otero SP, Metzger JW, Choi BH (2022). It’s time to deconstruct treatment-failure: a randomized controlled trial of nonoperative management of uncomplicated pediatric appendicitis with antibiotics alone. J Pediatr Surg.

[7] Collaborative CODA (2020). A randomized trial comparing antibiotics with appendectomy for Appendicitis. N Engl J Med.

[8] Salminen P, Paajanen H, Rautio T (2015). Antibiotic therapy vs appendectomy for treatment of uncomplicated Acute Appendicitis: the APPAC Randomized Clinical Trial. JAMA.

[9] Hall NJ, Eaton S, Sherratt FC (2021). CONservative TReatment of Appendicitis in Children: a randomised controlled feasibility trial (CONTRACT). Arch Dis Child.

[10] Humes DJ, Simpson J (2006). Acute appendicitis. BMJ.

[11] Wilms IM, de Hoog DE, de Visser DC, et al. Appendectomy versus antibiotic treatment for acute appendicitis Cochrane Database Syst Rev. 2011;(11):CD008359. 10.1002/14651858.CD008359.pub2. 10.1002/14651858.CD008359.pub2 22071846

[12] Ming PC, Yan TY, Tat LH (2009). Risk factors of postoperative infections in adults with complicated appendicitis. Surg Laparosc Endosc Percutan Tech.

[13] Konstantinidis KM, Anastasakou KA, Vorias MN (2008). A decade of laparoscopic appendectomy: presentation of 1026 patients with suspected appendicitis treated in a single surgical department. J Laparoendosc Adv Surg Tech A.

[14] NHS Appendicitis. 2019. Available online: https://www.nhs.uk/conditions/appendicitis/ (Accessed on 12 Feb 2021).

[15] Coldrey E (1959). Five years conservative treatment of acute appendicitis. J Int Coll Surg.

[16] Ditillo MF, Dziura JD, Rabinovici R (2006). Is it safe to delay appendectomy in adults with acute appendicitis?. Ann Surg.

[17] Andersson RE (2007). The natural history and traditional management of appendicitis revisited: spontaneous resolution and predominance of prehospital perforations imply that a correct diagnosis is more important than an early diagnosis. World J Surg.

[18] Livingston EH, Fomby TB, Woodward WA (2011). Epidemiological similarities between appendicitis and diverticulitis suggesting a common underlying pathogenesis. Arch Surg.

[19] Sallinen V, Akl EA, You JJ (2016). Meta-analysis of antibiotics versus appendicectomy for non-perforated acute appendicitis. Br J Surg.

[20] Harnoss JC, Zelienka I, Probst P (2017). Antibiotics versus surgical therapy for uncomplicated appendicitis: systematic review and meta-analysis of controlled trials (PROSPERO 2015:CRD42015016882). Ann Surg.

[21] Poprom N, Numthavaj P, Wilasrusmee C (2019). The efficacy of antibiotic treatment versus surgical treatment of uncomplicated acute appendicitis: systematic review and network meta-analysis of randomized controlled trial. Am J Surg.

[22] Rollins KE, Varadhan KK, Neal KR (2016). Antibiotics Versus Appendicectomy for the treatment of uncomplicated Acute Appendicitis: an updated Meta-analysis of Randomised controlled trials. World J Surg.

[23] Vons C, Barry C, Maitre S (2011). Amoxicillin plus clavulanic acid versus appendicectomy for treatment of acute uncomplicated appendicitis: an open-label, non-inferiority, randomised controlled trial. Lancet.

[24] Salminen P, Tuominen R, Paajanen H (2018). Five-year follow-up of antibiotic therapy for uncomplicated Acute Appendicitis in the APPAC Randomized Clinical Trial. JAMA.

[25] Patkova B, Svenningsson A, Almström M (2020). Nonoperative treatment Versus Appendectomy for Acute Nonperforated Appendicitis in Children: five-year follow up of a Randomized Controlled Pilot Trial. Ann Surg.

[26] Styrud J, Eriksson S, Nilsson I (2006). Appendectomy versus antibiotic treatment in acute appendicitis. A prospective multicenter randomized controlled trial. World J Surg.

[27] Bom WJ, Scheijmans JCG, Gans SL (2021). Population preference for treatment of uncomplicated appendicitis. BJS Open.

[28] Antoniou SA, Mavridis D, Kontouli KM (2021). EAES rapid guideline: appendicitis in the elderly. Surg Endosc.

[29] Wu S, Chen WT, Muo C (2015). Association between appendectomy and subsequent colorectal cancer development: an asian population study. PLoS ONE.

[30] Köhler F, Müller S, Hendricks A (2021). Changes in appendicitis treatment during the COVID-19 pandemic-A systematic review and meta-analysis. Int J Surg.

[31] Podda M, Cillara N, Di Saverio S, ACOI (Italian Society of Hospital Surgeons) Study Group on Acute Appendicitis (2017). Antibiotics-first strategy for uncomplicated acute appendicitis in adults is associated with increased rates of peritonitis at surgery. A systematic review with meta-analysis of randomized controlled trials comparing appendectomy and non-operative management with antibiotics. Surgeon.

[32] Podda M, Gerardi C, Cillara N (2019). Antibiotic treatment and appendectomy for uncomplicated Acute Appendicitis in adults and children: a systematic review and Meta-analysis. Ann Surg.

[33] Fugazzola P, Ceresoli M, Agnoletti V (2020). The SIFIPAC/WSES/SICG/SIMEU guidelines for diagnosis and treatment of acute appendicitis in the elderly (2019 edition). World J E Surg.

[34] Collard MK, Christou N, Lakkis Z (2021). Adult appendicitis: clinical practice guidelines from the French Society of Digestive surgery and the Society of Abdominal and Digestive Imaging. J Visc Surg.

[35] O’Leary DP, Walsh SM, Bolger J (2021). A Randomized Clinical Trial evaluating the efficacy and quality of life of antibiotic-only treatment of Acute uncomplicated appendicitis: results of the COMMA trial. Ann Surg.

[36] Sajjad MN, Naumeri F, Hina S (2021). Non-operative treatment versus appendectomy for acute uncomplicated appendicitis: a randomized controlled trial. Pak J Med Sci.

[37] Prechal D, Damirov F, Grilli M (2019). Antibiotic therapy for acute uncomplicated appendicitis: a systematic review and meta-analysis. Int J Colorectal Dis.

[38] Yang Z, Sun F, Ai S (2019). Meta-analysis of studies comparing conservative treatment with antibiotics and appendectomy for acute appendicitis in the adult. BMC Surg.

[39] Liberati A, Altman DG, Tetzlaff J (2009). The PRISMA statement for reporting systematic reviews and meta-analyses of studies that evaluate healthcare interventions: explanation and elaboration. BMJ.

[40] Higgins JPT, Green S. Cochrane Handbook for Systematic Reviews of Interventions Version 5.3.5. The Cochrane Collaboration, 2014. Available at www.handbook.cochrane.org. Accessed August 2018.

[41] Hansson J, Ko¨rner U, Khorram-Manesh A (2009). Randomized clinical trial of antibiotic therapy versus appendicectomy as primary treatment of acute appendicitis in unselected patients. Br J Surg.

[42] Turhan AN, Kapan S, Kütükçü E (2009). Comparison of operative and non operative management of acute appendicitis. Ulus Travma Acil Cerrahi Derg.

[43] Malik AA, Bari SU (2009). Conservative management of acute appendicitis. J Gastrointest Surg.

[44] Talan DA, Saltzman DJ, Mower WR (2017). Antibiotics-first Versus surgery for Appendicitis: a US pilot randomized controlled trial allowing Outpatient Antibiotic Management. Ann Emerg Med.

[45] Ceresoli M, Pisano M, Allievi N (2019). Never put equipoise in appendix! Final results of ASAA (antibiotics vs. surgery for uncomplicated acute appendicitis in adults) randomized controlled trial. Updates Surg.

[46] Svensson JF, PatkovaB, AlmströmM (2015). Nonoperative treatment with antibiotics versus surgery for acute nonperforated appendicitis in children: a pilot randomized controlled trial. Ann Surg.

[47] Eriksson S, Granström L (1995). Randomized controlled trial of appendicectomy versus antibiotic therapy for acute appendicitis. Br J Surg.

[48] Sippola S, Grönroos J, Tuominen R (2017). Economic evaluation of antibiotic therapy versus appendicectomy for the treatment of uncomplicated acute appendicitis from the APPAC randomized clinical trial. Br J Surg.

[49] Sippola S, Haijanen J, Viinikainen L (2020). Quality of life and patient satisfaction at 7-Year follow-up of antibiotic therapy vs appendectomy for uncomplicated Acute Appendicitis: a secondary analysis of a Randomized Clinical Trial. JAMA Surg.

[50] Haijanen J, Sippola S, Tuominen R (2019). Cost analysis of antibiotic therapy versus appendectomy for treatment of uncomplicated acute appendicitis: 5-year results of the APPAC randomized clinical trial. PLoS ONE.

[51] Luo D, Wan X, Liu J (2018). Optimally estimating the sample mean from the sample size, median, mid-range, and/or mid-quartile range. Stat Methods Med Res.

[52] Wan X, Wang W, Liu J (2014). Estimating the sample mean and standard deviation from the sample size, median, range and/or interquartile range. BMC Med Res Methodol.

[53] Emile SH, Sakr A, Shalaby M (2022). Efficacy and safety of non-operative management of uncomplicated Acute Appendicitis compared to appendectomy: an Umbrella Review of systematic reviews and Meta-analyses. World J Surg.

[54] FlumD R (2015). Acute appendicitis–appendectomy or the “antibiotics first” strategy. N Engl J Med.

[55] Andersson RE (2014). Short-term complications and long-term morbidity of laparoscopic and open appendicectomy in a national cohort. Br J Surg.

[56] Papandria D, Lardaro T, Rhee D (2013). Risk factors for conversion from laparoscopicto open surgery: analysis of 2138 converted operations in the American College of Surgeons National Surgical Quality Improvement Program. Am Surg.

[57] Childers CP, Dworsky JQ, Maggard-Gibbons M (2019). The contemporary appendectomy for acute uncomplicated appendicitis in adults. Surgery.

[58] Minneci PC, Mahida JB, Lodwick DL (2016). Effectiveness of patient choice in nonoperative vs Surgical Management of Pediatric Uncomplicated Acute Appendicitis. JAMA Surg.

[59] Ceresoli M, Coccolini F, Magnone S (2021). The decrease of non-complicated acute appendicitis andthe negative appendectomy rate during pandemic. Eur J Trauma Emerg Surg.

[60] Di Talan DA (2021). Treatment of Acute Uncomplicated Appendicitis. NEngl J Med.

[61] Flum DR, Koepsell T (2002). The clinical and economic correlates of misdiagnosed appendicitis: nationwide analysis. Arch Surg.

[62] Brockman SF, Scott S, Guest GD (2013). Does an Acute Surgical Model increase the rate of negative appendicectomy or perforated appendicitis?. ANZ J Surg.

[63] van Randen A, Bipat S, Zwinderman AH (2008). Acute appendicitis: Meta-analysis of diagnostic performance of CT and graded compression US related to prevalence of disease. Radiology.

[64] Donlon NE, Kelly ME, Sheppard A (2021). Negative appendicectomy rates as a quality measure in a regional surgical unit: a retrospective review. Ir J Med Sc.

[65] Minneci PC, Hade EM, GilL A (2022). Demographic and clinical characteristics Associated with the failure of Nonoperative Management of uncomplicated appendicitis in children: secondary analysis of a Nonrandomized Clinical Trial. JAMA Netw Open.

[66] Duke E, Kalb B, Arif-Tiwari H (2016). A systematic review and Meta-analysis of diagnostic performance of MRI for evaluation of Acute Appendicitis. AJR Am J Roentgenol.

[67] Varadhan KK, Neal KR, Lobo DN (2012). Safety and efficacy of antibiotics compared with appendicectomy for treatment of uncomplicated acute appendicitis: meta-analysis of randomised controlled trials. BMJ.

[68] Huang L, Yin Y, Yang L (2017). Comparison of antibiotic therapy and appendectomy for Acute uncomplicated appendicitis in children: a Meta-analysis. JAMA Pediatr.

[69] Podda M, Poillucci G, Pacella D (2021). Appendectomy versus conservative treatment with antibiotics for patients with uncomplicated acute appendicitis: a propensity score-matched analysis of patient-centered outcomes (the ACTUAA prospective multicenter trial). Int J Colorectal Dis.

[70] Shindoh J, Niwa H, Kawai K (2010). Predictive factors for negative outcomes in initial non-operative management of suspected appendicitis. J Gastrointest Surg.

[71] Mahida JB, Lodwick DL, Nacion KM (2016). High failure rate of nonoperative management of acute appendicitis with an appendicolith in children. J Pediatr Surg.

[72] Kohga A, Kawabe A, Yajima K (2021). Does the presence of an appendicolith or abscess predict failure of nonoperative management of patients with acute appendicitis?. Emerg Radiol.

[73] Lu P, McCarty JC, Fields AC (2019). Risk of appendiceal cancer in patients undergoing appendectomy for appendicitis in the era of increasing nonoperative management. J Surg Oncol.

[74] Charfi S, Sellami A, Affes A (2014). Histopathological findings in appendectomy specimens: a study of 24,697 cases. Int J Color Dis.

[75] Marudanayagam R, Williams GT, Rees BI (2006). Review of the pathological results of 2660 appendicectomy specimens. J Gastroenterol.

[76] Connor SJ, Hanna GB, FrizelleFA (1998). Appendiceal tumors: retrospective clinic opathologic analysis of appendiceal tumors from 7,970 appendectomies. Dis Colon Rectum.

[77] Loftus TJ, Raymond SL, Sarosi GA (2017). Predicting appendiceal tumors among patients with appendicitis. J Trauma Acute Care Surg.

[78] Furman MJ, Cahan M, Cohen P (2013). Increased risk of mucinous neoplasm of the appendix in adults undergoing interval appendectomy. JAMA Surg.

[79] Lietzén E, Grönroos JM, MecklinJP (2019). Appendiceal neoplasm risk associated with complicated cute appendicitis-a population based study. Int J Color Dis.

[80] Di Saverio S, Sibilio A, Giorgini E (2014). The NOTA study(non Operative Treatment for Acute Appendicitis): prospective study on the efficacy and safety of antibiotics (amoxicillin and clavulanic acid) for treating patients with right lower quadrant abdominal pain and long-term follow-up of conservatively treated suspected appendicitis. Ann Surg.

[81] Poillucci G, Mortola L, Podda M (2017). Laparoscopic appendectomy vs antibiotic therapy for acute appendicitis: a propensity score-matched analysis from a multicenter cohort study. Updates Surg.

