# Supplementary material for: Comparison of the efficacy and safety of antibiotic treatment and appendectomy for acute uncomplicated appendicitis: a systematic review and meta-analysis
Source: BMC Surg. 2023 Jul 24;23:208. doi: 10.1186/s12893-023-02108-1 (PMC10367319; doi:10.1186/s12893-023-02108-1)
Supplement: Supplementary file 1 — Additional file 1. [file 12893_2023_2108_MOESM1_ESM.docx]

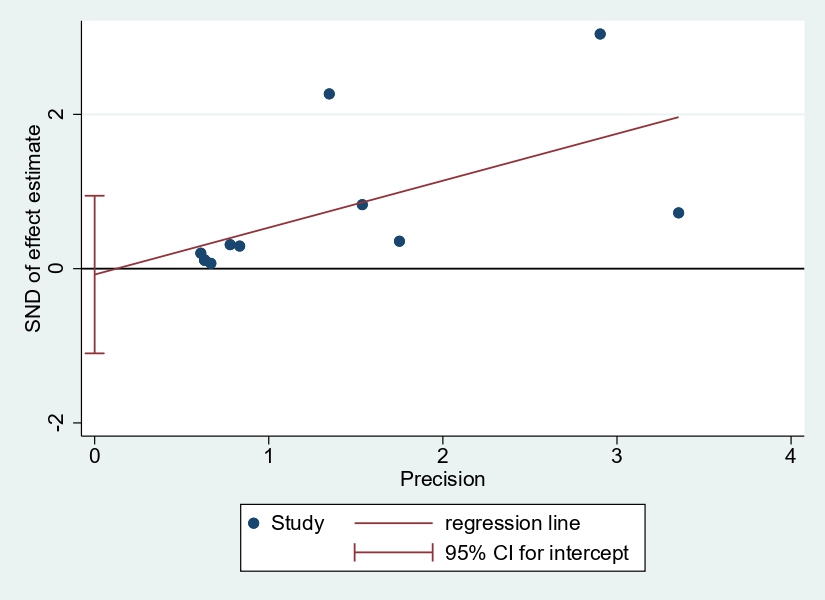


Annex 1. Egger diagram of complications


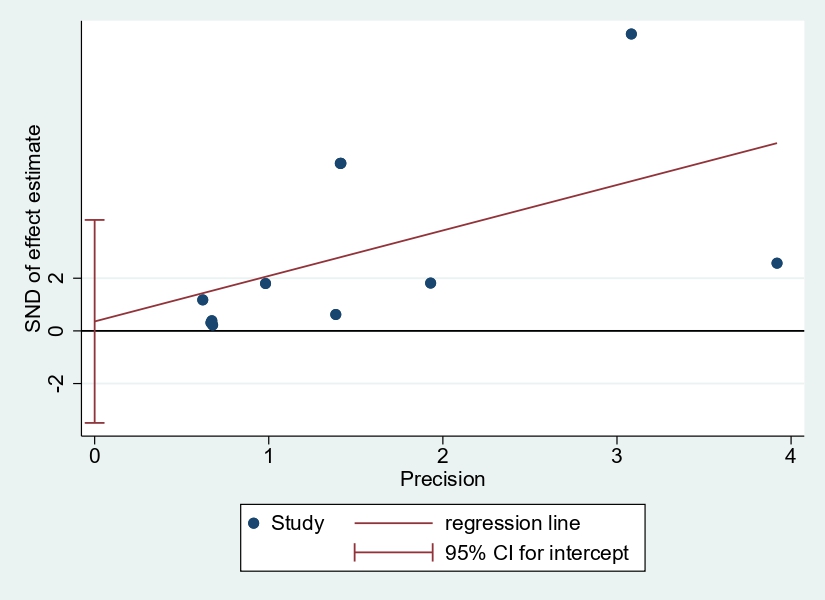


Annex 2. Egger diagram of postoperative complications


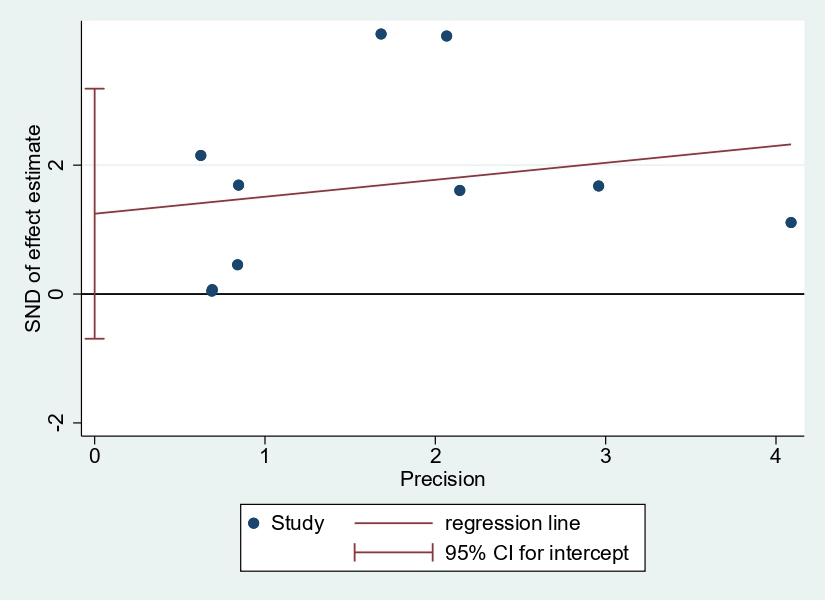


Annex 3. Egger diagram of the complicated appendicitis rate


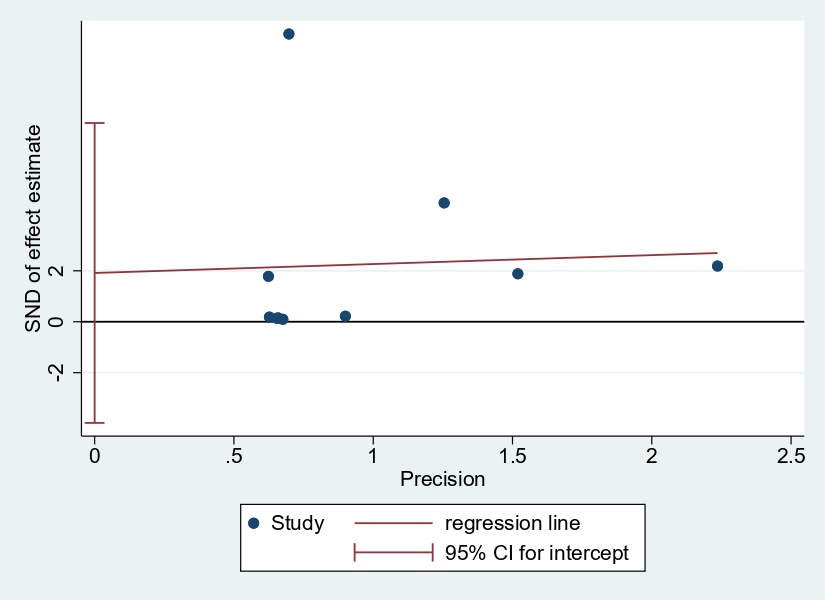


Annex 4. Egger diagram of negative appendicectomies


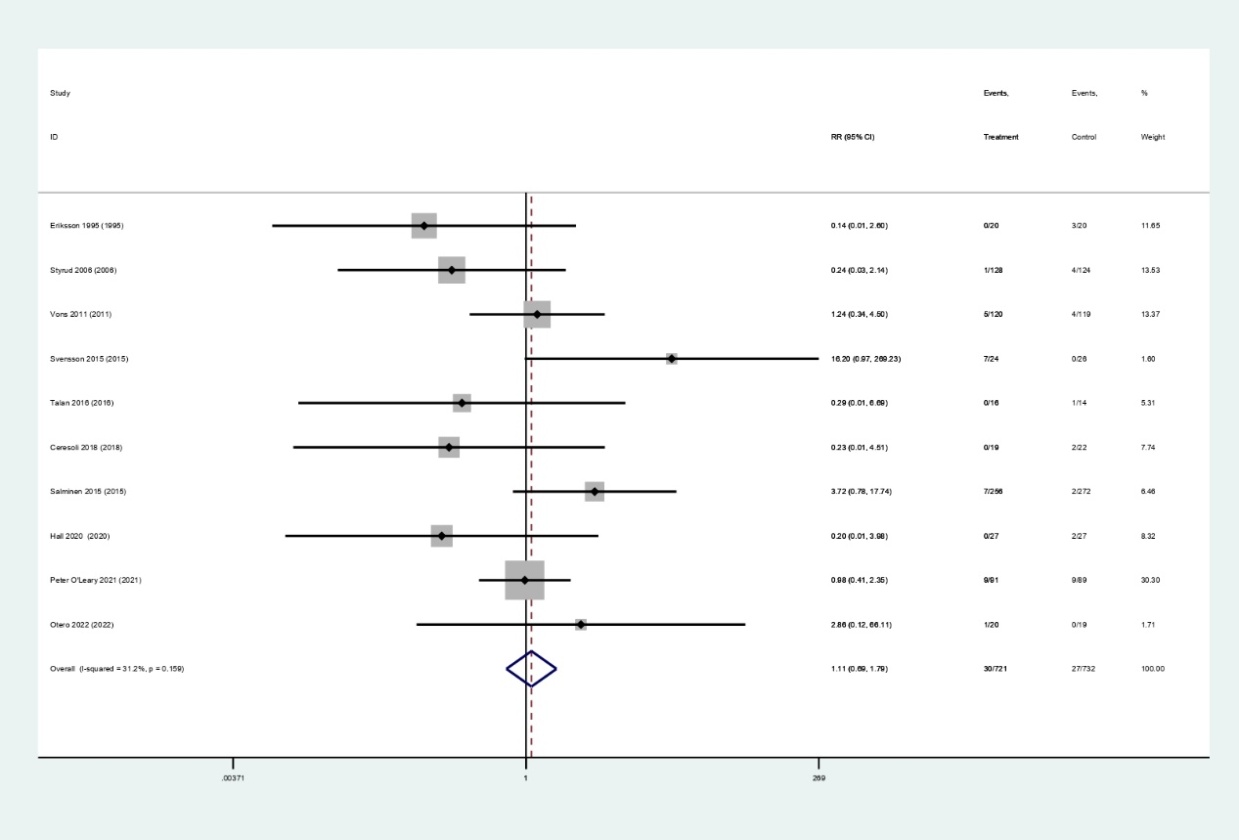


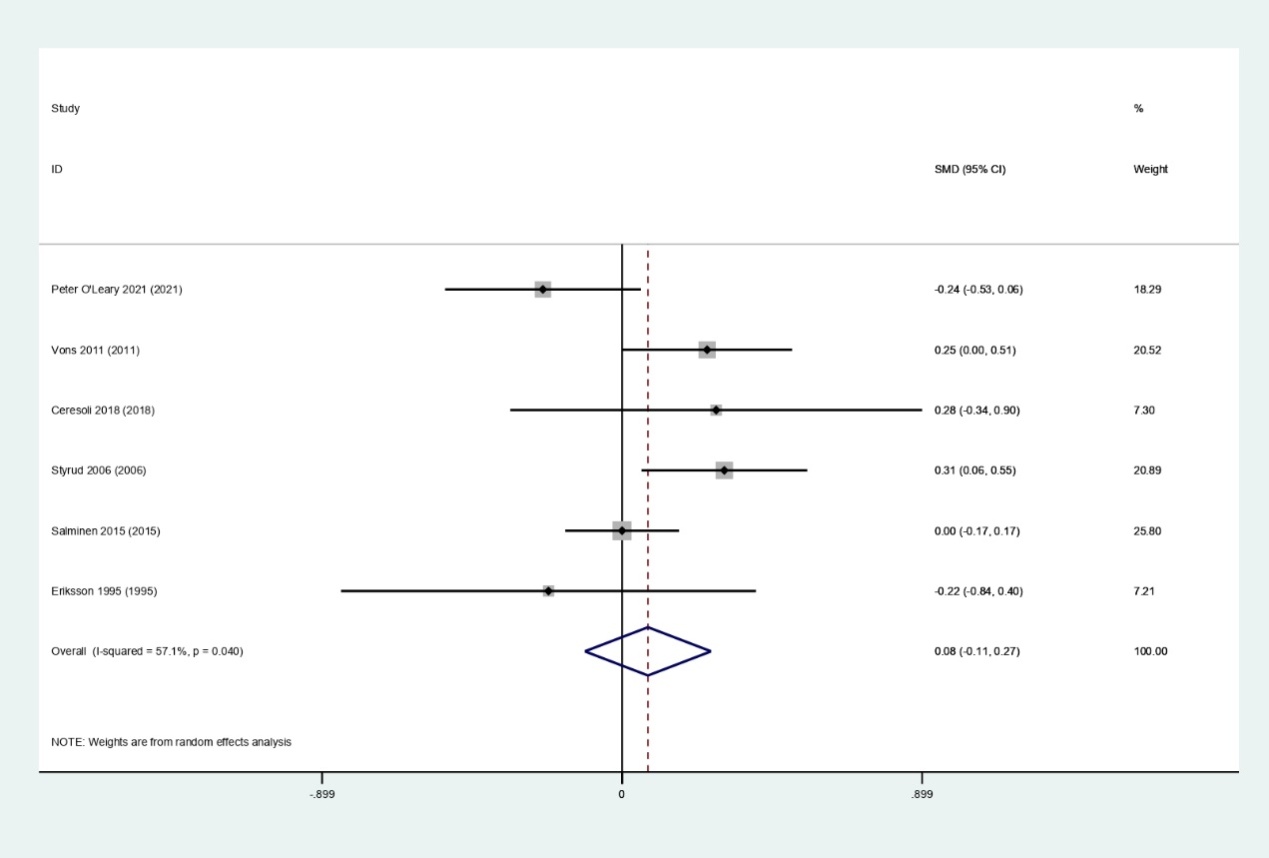
Annex 5. Forest diagram of negative appendicectomies

Annex 6. Forest diagram of hospitalization time


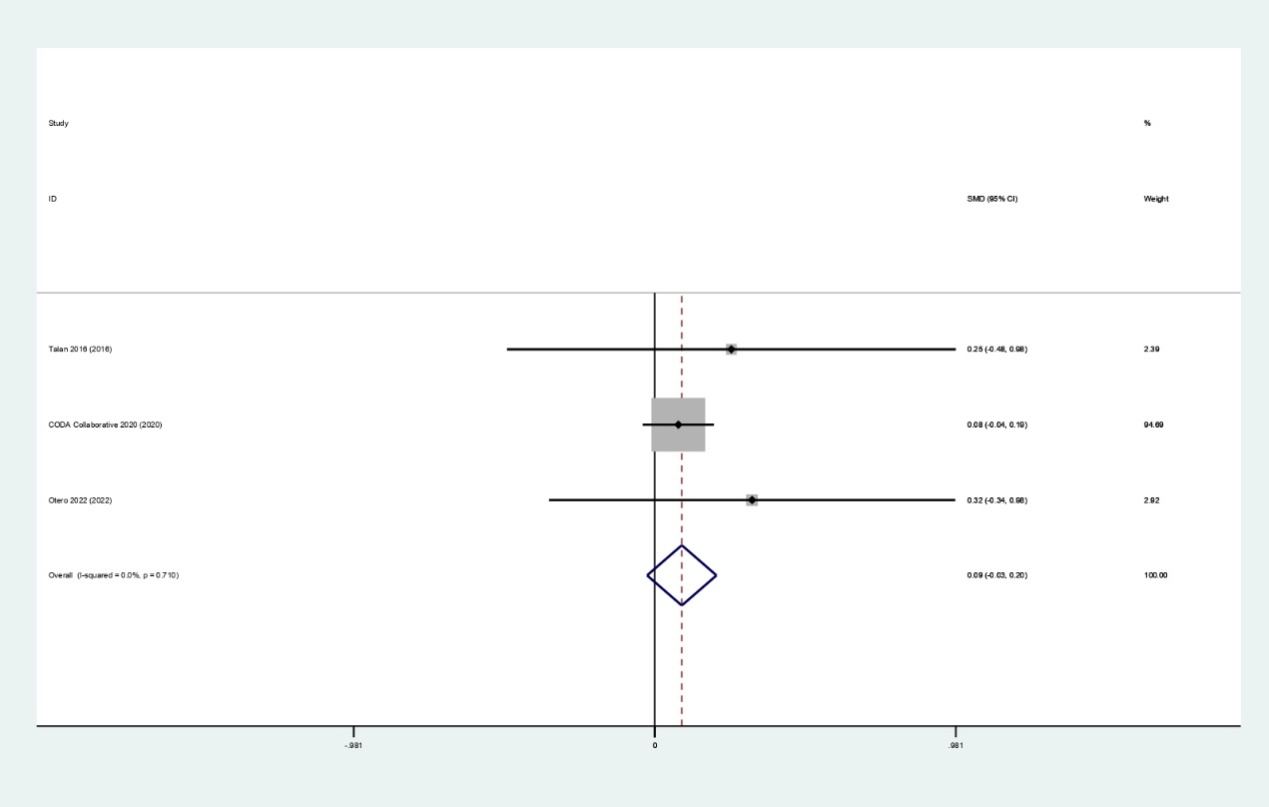


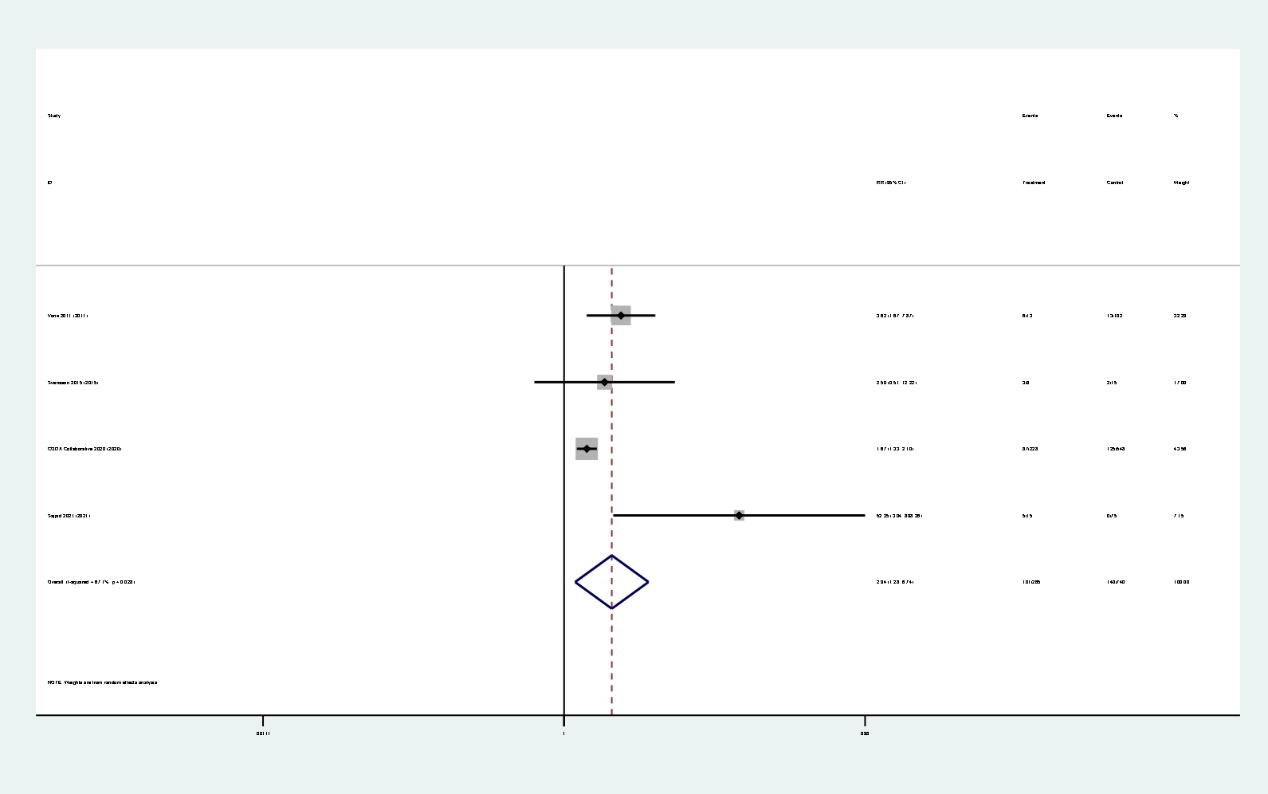
Annex 7. Forest diagram of 1-month quality of life

Annex 8. Forest diagram of the influence of appendix stones on antibiotic treatment

Supplementary Table 1: The search strategies of English database

| database | search strategy |
| --- | --- |
| PubMed | (((("Appendicitis"[Mesh]) OR ((((Ruptured Appendicitis[Title/Abstract]) OR (Appendicitis, Ruptured[Title/Abstract])) OR (Perforated Appendicitis[Title/Abstract])) OR (Appendicitis, Perforated[Title/Abstract]))) AND (("Appendectomy"[Mesh]) OR (Appendectomies[Title/Abstract]))) AND (("Anti-Bacterial Agents"[Mesh]) OR (((((((((((((((((((((((((((((((((Agents, Anti-Bacterial[Title/Abstract]) OR (Anti Bacterial Agents[Title/Abstract])) OR (Antibacterial Agents[Title/Abstract])) OR (Agents, Antibacterial[Title/Abstract])) OR (Antibacterial Agent[Title/Abstract])) OR (Agent, Antibacterial[Title/Abstract])) OR (Anti-Bacterial Compounds[Title/Abstract])) OR (Anti Bacterial Compounds[Title/Abstract])) OR (Compounds, Anti-Bacterial[Title/Abstract])) OR (Anti-Bacterial Agent[Title/Abstract])) OR (Agent, Anti-Bacterial[Title/Abstract])) OR (Anti Bacterial Agent[Title/Abstract])) OR (Anti-Bacterial Compound[Title/Abstract])) OR (Anti Bacterial Compound[Title/Abstract])) OR (Compound, Anti-Bacterial[Title/Abstract])) OR (Bacteriocidal Agents[Title/Abstract])) OR (Agents, Bacteriocidal[Title/Abstract])) OR (Bacteriocidal Agent[Title/Abstract])) OR (Agent, Bacteriocidal[Title/Abstract])) OR (Bacteriocide[Title/Abstract])) OR (Bacteriocides[Title/Abstract])) OR (Anti-Mycobacterial Agents[Title/Abstract])) OR (Agents, Anti-Mycobacterial[Title/Abstract])) OR (Anti Mycobacterial Agents[Title/Abstract])) OR (Anti-Mycobacterial Agent[Title/Abstract])) OR (Agent, Anti-Mycobacterial[Title/Abstract])) OR (Anti Mycobacterial Agent[Title/Abstract])) OR (Antimycobacterial Agent[Title/Abstract])) OR (Agent, Antimycobacterial[Title/Abstract])) OR (Antimycobacterial Agents[Title/Abstract])) OR (Agents, Antimycobacterial[Title/Abstract])) OR (Antibiotics[Title/Abstract])) OR (Antibiotic[Title/Abstract])))) AND (("Randomized Controlled Trial" [Publication Type]) OR (rct AND (1000/1/1:2022/5/31[pdat])) AND (1000/1/1:2022/5/31[pdat])) Filters: from 1000/1/1 - 2022/5/31. |
| Embase | #14. #3 AND #6 AND #9 AND #12  #13. #3 AND #6 AND #9 AND #12  #12. #10 OR #11  #11. ('randomized controlled trial (topic)'/exp OR 'randomized controlled trial (topic)' OR (randomized AND controlled AND trial) OR rct) AND [01-01-2022]/sd NOT [01-06-2022]/sd  #10. ('randomized controlled trial (topic)'/exp OR 'randomized controlled trial (topic)' OR (randomized AND controlled AND trial) OR rct) AND [<1966-2021]/py  #9. #7 OR #8  #8. ('anti-bacterial agents'/exp OR 'anti-bacterial agents' OR ('anti bacterial' AND agents) OR 'agents, anti-bacterial':ti,ab,kw OR 'anti bacterial agents':ti,ab,kw OR 'antibacterial agents':ti,ab,kw OR 'agents, antibacterial':ti,ab,kw OR 'antibacterial agent':ti,ab,kw OR 'agent, antibacterial':ti,ab,kw OR 'anti-bacterial  compounds':ti,ab,kw OR 'anti bacterial compounds':ti,ab,kw OR 'compounds, anti-bacterial':ti,ab,kw OR 'anti-bacterial agent':ti,ab,kw OR 'agent, anti-bacterial':ti,ab,kw OR 'anti bacterial agent':ti,ab,kw OR 'anti-bacterial compound':ti,ab,kw OR 'anti bacterial compound':ti,ab,kw OR 'compound, anti-bacterial':ti,ab,kw OR 'bacteriocidal  agents':ti,ab,kw OR 'agents, bacteriocidal':ti,ab,kw OR 'bacteriocidal agent':ti,ab,kw OR 'agent, bacteriocidal':ti,ab,kw OR bacteriocide:ti,ab,kw OR bacteriocides:ti,ab,kw OR 'anti-mycobacterial agents':ti,ab,kw OR 'agents, anti-mycobacterial':ti,ab,kw OR 'anti mycobacterial agents':ti,ab,kw OR 'anti-mycobacterial agent':ti,ab,kw OR 'agent,anti-mycobacterial':ti,ab,kw OR 'anti mycobacterial agent':ti,ab,kw OR 'antimycobacterial agent':ti,ab,kw OR 'agent, antimycobacterial':ti,ab,kw OR 'antimycobacterial agents':ti,ab,kw OR 'agents, antimycobacterial':ti,ab,kw OR  antibiotics:ti,ab,kw OR antibiotic:ti,ab,kw) AND [01-01-2022]/sd NOT [01-06-2022]/sd  #7. ('anti-bacterial agents'/exp OR 'anti-bacterial agents' OR ('anti bacterial' AND agents) OR 'agents, anti-bacterial':ti,ab,kw OR 'anti bacterial agents':ti,ab,kw OR 'antibacterial agents':ti,ab,kw OR 'agents, antibacterial':ti,ab,kw OR 'antibacterial agent':ti,ab,kw OR 'agent, antibacterial':ti,ab,kw OR 'anti-bacterial compounds':ti,ab,kw OR 'anti bacterial compounds':ti,ab,kw OR 'compounds, anti-bacterial':ti,ab,kw OR 'anti-bacterial  agent':ti,ab,kw OR 'agent, anti-bacterial':ti,ab,kw OR 'anti bacterial agent':ti,ab,kw OR 'anti-bacterial compound':ti,ab,kw OR 'anti bacterial compound':ti,ab,kw OR 'compound, anti-bacterial':ti,ab,kw OR 'bacteriocidal  agents':ti,ab,kw OR 'agents, bacteriocidal':ti,ab,kw OR 'bacteriocidal agent':ti,ab,kw OR 'agent, bacteriocidal':ti,ab,kw OR bacteriocide:ti,ab,kw OR bacteriocides:ti,ab,kw OR 'anti-mycobacterial agents':ti,ab,kw OR 'agents, anti-mycobacterial':ti,ab,kw OR 'anti mycobacterial agents':ti,ab,kw OR 'anti-mycobacterial agent':ti,ab,kw OR 'agent, anti-mycobacterial':ti,ab,kw OR 'anti mycobacterial agent':ti,ab,kw OR 'antimycobacterial agent':ti,ab,kw OR 'agent, antimycobacterial':ti,ab,kw OR 'antimycobacterial agents':ti,ab,kw OR 'agents, antimycobacterial':ti,ab,kw OR antibiotics:ti,ab,kw OR antibiotic:ti,ab,kw) AND [<1966-2021]/py  #6. #4 OR #5  #5. ('appendectomy'/exp OR appendectomy OR appendectomies:ti,ab,kw) AND [01-01-2022]/sd NOT [01-06-2022]/sd  #4. ('appendectomy'/exp OR appendectomy OR appendectomies:ti,ab,kw) AND [<1966-2021]/py  #3. #1 OR #2  #2. ('acute appendicitis'/exp OR 'acute appendicitis' OR 'ruptured appendicitis':ti,ab,kw OR 'appendicitis, ruptured':ti,ab,kw OR 'perforated appendicitis':ti,ab,kw OR 'appendicitis, perforated':ti,ab,kw) AND [01-01-2022]/sd NOT [01-06-2022]/sd  #1. ('acute appendicitis'/exp OR 'acute appendicitis' OR 'ruptured appendicitis':ti,ab,kw OR 'appendicitis, ruptured':ti,ab,kw OR 'perforated appendicitis':ti,ab,kw OR 'appendicitis, perforated':ti,ab,kw) AND [<1966-2021]/py |
| Cochrane | #1 MeSH descriptor: [Appendicitis] explode all trees  #2 (Ruptured Appendicitis):ti,ab,kw OR(Appendicitis, Ruptured):ti,ab,kw OR (Perforated Appendicitis):ti,ab,kw OR (Appendicitis, Perforated):ti,ab,kw (Word variations have been searched)  #3 #1 OR #2  #4 MeSH descriptor: [Appendectomy] explode all trees  #5 (Appendectomies):ti,ab,kw (Word variations have been searched)  #6 #4 OR #5  #7 MeSH descriptor: [Anti-Bacterial Agents] explode all trees  #8 (Agents, Anti-Bacterial):ti,ab,kw OR (Anti Bacterial Agents):ti,ab,kw OR (Antibacterial Agents):ti,ab,kw OR (Agents, Antibacterial):ti,ab,kw OR (Antibacterial Agent):ti,ab,kw (Word variations have been searched)  #9 (Agent, Antibacterial):ti,ab,kw OR (Anti-Bacterial Compounds):ti,ab,kw OR (Anti Bacterial Compounds):ti,ab,kw OR (Compounds, Anti-Bacterial):ti,ab,kw OR (Anti-Bacterial Agent):ti,ab,kw (Word variations have been searched)  #10 (Agent, Anti-Bacterial):ti,ab,kw OR (Anti Bacterial Agent):ti,ab,kw OR (Anti-Bacterial Compound):ti,ab,kw OR (Anti Bacterial Compound):ti,ab,kw OR (Compound, Anti-Bacterial):ti,ab,kw (Word variations have been searched)  #11 (Bacteriocidal Agents):ti,ab,kw OR (Agents, Bacteriocidal):ti,ab,kw OR (Bacteriocidal Agent):ti,ab,kw OR (Agent, Bacteriocidal):ti,ab,kw OR (Bacteriocide):ti,ab,kw (Word variations have been searched)  #12 (Bacteriocides):ti,ab,kw OR (Anti-Mycobacterial Agents):ti,ab,kw OR (Agents, Anti-Mycobacterial):ti,ab,kw OR (Anti Mycobacterial Agents):ti,ab,kw OR (Anti-Mycobacterial Agent):ti,ab,kw (Word variations have been searched)  #13 (Agent, Anti-Mycobacterial):ti,ab,kw OR (Anti Mycobacterial Agent):ti,ab,kw OR (Antimycobacterial Agent):ti,ab,kw OR (Agent, Antimycobacterial):ti,ab,kw OR (Antimycobacterial Agents):ti,ab,kw (Word variations have been searched)  #14 (Agents, Antimycobacterial):ti,ab,kw OR (Antibiotics):ti,ab,kw OR (Antibiotic):ti,ab,kw (Word variations have been searched)  #15 #7 OR #8 OR #9 OR #10 OR #11 OR #12 OR #13 OR #14  #16 (Randomized Controlled Trial) OR (RCT) (Word variations have been searched)  #17 #3 AND #6 AND #15 AND #16 |
| Web of Science | 1: ((((TS=(Appendicitis)) OR TS=(Ruptured Appendicitis)) OR TS=(Appendicitis, Ruptured)) OR TS=(Perforated Appendicitis)) OR TS=(Appendicitis, Perforated)  2: (TS=(Appendectomy)) OR TS=(Appendectomies)  3: (((((((((((((((((((((((((((((((((TS=(Anti-Bacterial Agents)) OR TS=(Agents, Anti-Bacterial)) OR TS=(Anti Bacterial Agents)) OR TS=(Antibacterial Agents)) OR TS=(Agents, Antibacterial)) OR TS=(Antibacterial Agent)) OR TS=(Agent, Antibacterial)) OR TS=(Anti-Bacterial Compounds)) OR TS=(Anti Bacterial Compounds)) OR TS=(Compounds, Anti-Bacterial)) OR TS=(Anti-Bacterial Agent)) OR TS=(Agent, Anti-Bacterial)) OR TS=(Anti Bacterial Agent)) OR TS=(Anti-Bacterial Compound)) OR TS=(Anti Bacterial Compound)) OR TS=(Compound, Anti-Bacterial)) OR TS=(Bacteriocidal Agents)) OR TS=(Agents, Bacteriocidal)) OR TS=(Bacteriocidal Agent)) OR TS=(Agent, Bacteriocidal)) OR TS=(Bacteriocide)) OR TS=(Bacteriocides)) OR TS=(Anti-Mycobacterial Agents)) OR TS=(Agents, Anti-Mycobacterial)) OR TS=(Anti Mycobacterial Agents)) OR TS=(Anti-Mycobacterial Agent)) OR TS=(Agent, Anti-Mycobacterial)) OR TS=(Anti Mycobacterial Agent)) OR TS=(Antimycobacterial Agent)) OR TS=(Agent, Antimycobacterial)) OR TS=(Antimycobacterial Agents)) OR TS=(Agents, Antimycobacterial)) OR TS=(Antibiotics)) OR TS=(Antibiotic)  4: (ALL=(Randomized Controlled Trial)) OR ALL=(RCT)  5: #4 AND #3 AND #2 AND #1 |
